# Vanishing Lung Syndrome in a Patient with HIV Infection and Heavy Marijuana Use

**DOI:** 10.1155/2014/285208

**Published:** 2014-01-08

**Authors:** Basheer Tashtoush, Fernando Gonzalez-Ibarra, Roya Memarpour, Anas Hadeh, Laurence Smolley

**Affiliations:** ^1^Department of Pulmonary and Critical Care Medicine, Cleveland Clinic Florida, 2950 Cleveland Clinic Boulevard, Weston, FL 33331, USA; ^2^Department of Internal Medicine, Jersey City Medical Center, Mount Sinai School of Medicine, 355 Grand Street, Jersy City, NJ 07302, USA

## Abstract

Vanishing lung syndrome (VLS) is a rare and distinct clinical syndrome that usually affects young men. VLS leads to severe progressive dyspnea and is characterized by extensive, asymmetric, peripheral, and predominantly upper lobe giant lung bullae. Case reports have suggested an additive role of marijuana use in the development of this disease in young male tobacco smokers. We herein report a case of a 65-year-old Hispanic male previously diagnosed with severe emphysema and acquired immune deficiency syndrome (AIDS), with a history of intravenous heroin use and active marijuana smoking who presents to the emergency department with severe progressive shortness of breath he was found to have multiple large subpleural bullae occupying more than one-third of the hemithorax on chest computerized tomography (CT), characteristic of vanishing lung syndrome. The patient was mechanically ventilated and later developed a pneumothorax requiring chest tube placement and referral for surgical bullectomy. Surgical bullectomy has shown high success rates in alleviating the debilitating symptoms and preventing the life threatening complications of this rare syndrome. This case further emphasizes the importance of recognizing VLS in patients with severe emphysema and heavy marijuana smoking.

## 1. Introduction

Vanishing lung syndrome, also termed giant bullous emphysema (GBE), is a rare syndrome first described by Burke in 1937 [1]. It is an idiopathic and distinct clinical syndrome that affects young men, usually smokers. It causes severe progressive dyspnea and is characterized by extensive, predominantly asymmetric upper lobe bullous emphysema, which may eventually lead to respiratory failure [2]. Case reports have suggested an additive role of marijuana smoking in the development of this disease in young male smokers where tobacco consumption was less than what is commonly associated with the development of emphysema (i.e., less than 20 pack years) [3, 4].

We report a case of a 65-year-old male diagnosed with end stage emphysema for 3 years, AIDS, and a history of heavy marijuana smoking. He presents to the hospital in acute respiratory distress with multiple large peripheral lung bullae on chest CT consistent with vanishing lung syndrome.

## 2. Case Presentation

A 65-year-old Hispanic male was brought to the emergency department due to severe shortness of breath at home. The shortness of breath started approximately one week prior to presentation and had progressively worsened over time. Upon arrival to the emergency department the patient was hypoxemic and in severe respiratory distress, with minimal bilateral breath sounds on auscultation and an oxygen saturation of 88%.

He had a past medical history of severe chronic obstructive pulmonary disease (COPD), treated with home oxygen over the last year, history of AIDS for 17 years, receiving antiretroviral medications, (last documented HIV RNA load of 14,070 copies/mm^3^ and a CD_4_ count of 285/mm^3^). He had history of intravenous heroin use which he quit 6 years earlier, active heavy marijuana smoking for the last 20 years on a daily-basis, and no history of tobacco use.

**Figure 1:**
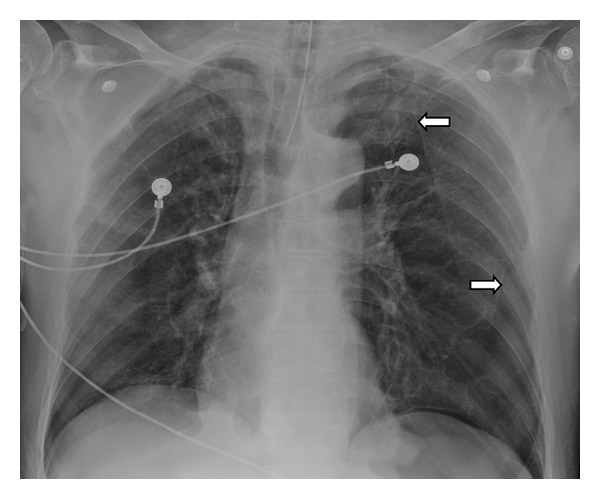
Anteroposterior chest X-ray following endotracheal tube placement showing hyperinflated lungs with multiple areas of hyperlucency on the left lung base and left apex, surrounded by a thin linear demarcation (arrows).

**Figure 2:**
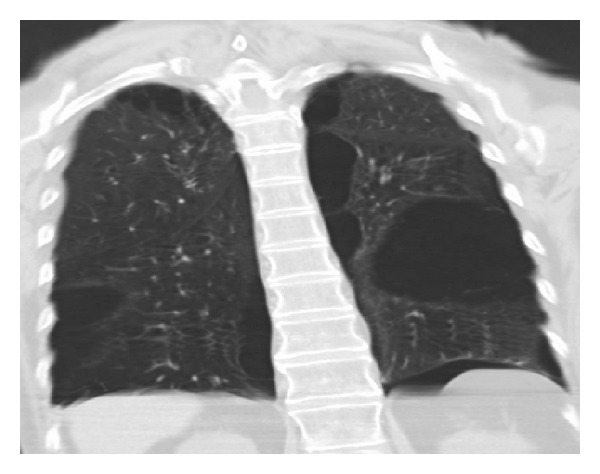
Chest CT coronal view showing bilateral lung bullae more than one-third of the left hemithorax.

**Figure 3:**
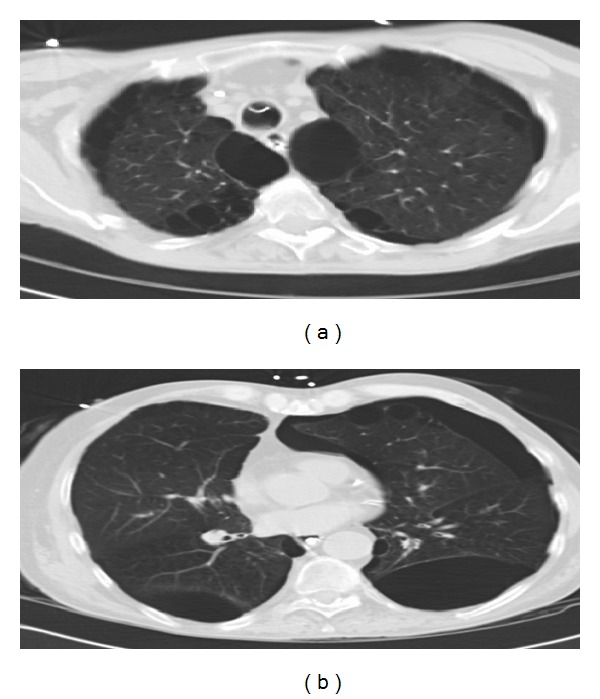
Chest CT axial view showing bilateral upper lobe (a) and lower lobe (b) bullae compressing surrounding relatively normal lung parenchyma.

On physical examination the patient was intubated on mechanical ventilation, with reduced bilateral breath sounds and minimal chest wall expansion. Arterial blood gas analysis showed respiratory acidosis with no other laboratory abnormalities. serum antineutrophil antibodies (ANA) were negative; serum angiotensin converting enzyme (ACE) and alpha 1 antitrypsine were at normal levels. Chest X-ray showed hyperinflation with increased lucency in bilateral upper lobes (Figure 1). Chest CT revealed multiple large subpleural bullae occupying more than one-third of the hemithorax, characteristic of VLS (Figures 2 and 3).

During the course of hospitalization, while on mechanical ventilation, the patient developed a tension pneumothorax secondary to ruptured bullae, requiring a chest tube placement. He was later referred for surgical bullectomy.

## 3. Discussion 

Bullae are described as air-filled spaces exceeding 1 cm in size with a wall thickness less than 1 mm. Giant bullae form when adjacent areas of paraseptal emphysema coalesce and thus are usually subpleural in distribution [5].

It was originally hypothesized that bullae formation was due to a “ball-valve mechanism” in which gas entered the lesion but could not escape. New evidence based on the measurement of intraluminal bulla pressure, however, suggests that there is negative intrabullous pressure, similar to pleural pressure, and is thus preferentially ventilated during inspiration, and with the considerable loss of elastic recoil air trapping occurs. This gradient between a bulla and the alveolar space provides an explanation for the radiographically and intraoperatively observed compression of lung tissue immediately adjacent to a bulla [6].

Several risk factors for the development of VLS have been described in literature, including tobacco smoking, marijuana smoking [3, 4, 7], alpha 1 antitrypsin deficiency [8], autoimmune and connective tissue diseases such as SLE, and sarcoidosis [9]. In subjects who smoke marijuana, the pathological changes of emphysema occur at a younger age (approximately 20 years earlier) than in tobacco smokers [3, 4]. These changes can also be distinguished from tobacco induced emphysema by the location of the large pulmonary bullae, found mostly in a paraseptal distribution with a marked predisposition for the upper lobes, as compared to the more uniformly distributed bullae of centrilobular emphysema which are the typical changes associated with a lifetime of tobacco smoking [7, 10, 11].

How marijuana might cause such severe bullous disease is not clear. It has been postulated that the methods of inhalation of marijuana smoke may cause significant barotrauma. Marijuana smokers tend to take much deeper breaths and employ breath-holding techniques for up to four times longer than cigarette smokers, with a nearly 70% increase in inspiratory volume. When this smoking practice is combined with the lack of filter tips on marijuana cigarettes, it leads to a fourfold greater delivery of tar and a five-time greater increase in carboxyhaemoglobin per cigarette smoked [12–14].

HIV infection also confers an increased risk for emphysema when compared to control subjects (15% versus 1%, resp.), with studies showing an accelerated progression towards emphysema in HIV-positive individuals who smoke [15]. Spirometry based studies revealed that HIV infection is an independent risk factor for the development of obstructive lung disease [16], which is thought to be secondary to the decreased antioxidant defenses, especially superoxide dismutase and glutathione in HIV-positive patients [17].

The radiographic criteria for vanishing lung syndrome are defined by the presence of giant bullae in one or both upper lobes, occupying at least one-third of the hemithorax and compressing surrounding normal lung parenchyma. Stern et al. described the CT findings of giant bullous emphysema, which included multiple large bullae, ranging from 1 to 20 cm in diameter without a single dominant giant bulla [18].

A major complication of VLS is pneumothorax, which classically presents as a history of acute deterioration in respiratory function associated with chest pain. When the diagnosis on chest radiography is uncertain, chest CT is recommended [19].

Treatment of VLS is usually surgical resection of the giant bullae, especially when patients are symptomatic or develop a pneumothorax. Surgical options include standard thoracotomy and video-assisted thoracoscopic surgery, with no apparent differences in outcomes between one procedure and the other [20]. Long term studies showed that lung resection for giant bullae in selected patients reduce the dynamic hyperinflation of the chest, with a significant improvement in the patients lung function and exercise performance [21].

## 4. Conclusion

This case emphasizes the importance of recognizing VLS in patients with history of heavy marijuana smoking, who present with findings of severe and atypical emphysema on imaging studies, as surgical bullectomy becomes a critical therapeutic option to prevent the life threatening complications of this rare syndrome.
